# Knee injuries in severe trauma patients: a trauma registry study in 3.458 patients

**DOI:** 10.1186/1752-2897-6-7

**Published:** 2012-08-06

**Authors:** Hagen Andruszkow, Emmanouil Liodakis, Rolf Lefering, Christian Krettek, Frank Hildebrand, Carl Haasper

**Affiliations:** 1 Trauma Department, Hannover Medical School, Carl-Neuberg-Straße 1, Hannover, 30625, Germany; 2 Institute for Research in Operative Medicine (IFOM), Cologne-Merheim Medical Center, University Witten-Herdecke, Cologne, Germany; 3 Committee on Emergency Medicine, Intensive and Trauma Care of the German Society for Trauma Surgery (DGU), Cologne, Germany

## Abstract

**Background:**

Purpose of the presented study is to answer the following questions: Are knee injuries associated with trauma mechanisms or concomitant injuries? Do injuries of the knee region aggravate treatment costs or prolong hospital stay in polytraumatized patients?

**Methods:**

A retrospective analysis including 29.779 severely injured patients (Injury Severity Score [greater than or equal to] 16) from the Trauma Registry of the German Society for Trauma Surgery database (1993-2008) was conducted. Patients were subdivided into two groups; the "Knee" group (n=3.458, 11.6% of all patients) including all multiple trauma patients with knee injuries, and the "Non Knee" group (n=26.321) including the remaining patients. Patients with knee injuries were slightly younger, less often male gender and had a significantly increased ISS.

**Results:**

Patients in the Knee group suffered significantly more traffic accidents compared to the Non Knee group (82% vs. 52%, p<0.001). These injuries were more often caused by car or motorbike accidents. Severe thoracic and limb injuries (AIS[greater than or equal to]3) were more frequently found in the Knee group (p<0.001) while head injury was distributed equally. The overall hospital stay, ICU stay, and treatment costs were significantly higher for the Knee group (38.1 vs. 25.5 days, 15.2 vs. 11.4 days, 40,116 vs. 25,336 Euro, respectively; all p<0.001).

**Conclusions:**

Traffic accidents are associated with an increased incidence of knee injuries than falls or attempted suicides. Furthermore, severe injuries of the limbs and chest are more common in polytraumatized patients with knee injuries. At last, treatment of these patients is prolonged and consequently more expensive.

## Background

Knee injuries are commonly described in multiple traumatized (“polytraumatized”) patients [1,2]. Although lower limb injuries and especially below knee injuries may not be life threatening apriori, they could cause significant functional disabilities with long lasting physical and psychosocial consequences [3-5]. Moreover, the increased risk of long-term sequels due to the complexity of knee injuries leads to relatively high financial costs [4,6]. Consequently, the socio-economic costs increase with the reduction of mortality of polytraumatized patients [7].

Despite the obvious relevance of lower limb injuries, they are commonly underestimated during the acute care setting while life threatening injuries or their complications such as sepsis or acute distress syndrome (ARDS) are treated [8]. To the best of our knowledge there are currently no studies designed to identify correlations between knee injuries and trauma mechanisms or concomitant injuries in patients suffering multiple trauma. This knowledge would lead physicians to focused search for knee injuries in high risk patients and may also influence the early initiation of treatment.

Therefore, the purpose of the presented study is to answer the following questions: (1) Do significant associations exist between knee injuries and trauma mechanisms or concomitant injuries? (2) Do injuries of the knee region aggravate treatment costs or prolong hospital stay in multiple trauma patients?

## Methods

### The Trauma Registry of the German Society for Trauma Surgery (TR-DGU)

The TR-DGU database is a multicenter, prospective, standardized and anonymous documentation of severely traumatized patients at four consecutive stages: A, pre-hospital phase; B, emergency room and initial therapy until admission to intensive care unit (ICU); C, ICU phase; D, discharge from acute care hospital and outcome. Until 2008, 145 hospitals were affiliated with the TR-DGU, mostly from Germany (n = 128) [9]. The database contains detailed information on demographics, injury pattern, co-morbidities, pre-clinical and clinical management, time course, relevant laboratory findings and outcome of each individual. All data are centrally evaluated for completeness and reliability before entering into the database [8]. Injuries are coded according to the Abbreviated Injury Scale (AIS) following its 2005 revised version. The Trauma Registry is approved by the review board of the German Trauma Society (Deutsche Gesellschaft für Unfallchirurgie, DGU).

### Inclusion and exclusion criteria/ Classification of knee injuries

All patients with an Injury Severity Score (ISS) ≥ 16 documented in the TR-DGU from 1993–2008 were included in the present study.

Knee injuries were defined as all bony injuries of the patella, as well as soft tissue injuries involving the knee region with or without the involvement of articular cartilage. Distal femoral and proximal tibial fractures were considered as knee injuries as well.

Severity of injuries was recorded using the Abbreviated Injury Scale (AIS) as 1 (minor), 2 (moderate), 3 (severe, not life threatening), 4 (serious, life threatening), 5 (critical, survival uncertain), 6 (maximum, currently untreatable) [10]. However, the severity of knee injuries ranges only from one to three [2,11].

### Statistics

Treatment costs were based on a modular cost estimator based on data from the TR-DGU [12]. Continuous values are presented as mean ± standard deviation (SD). Differences between the groups were evaluated with Student’s t-test for continuous data, while Pearson’s Chi-Square test was used for categorical values. A p-value ≤ 0.01 (two tailed) was considered to be statistically significant. Due to the large sample size statistical significance does not necessary imply clinical relevance. All statistical analyses were performed using SPSS (SPSS 18.0, IBM Inc., Somers, NY, USA).

## Results

29.779 patients were included in the presented study and subsequently divided into two groups according to the presence of a knee injury. The group “Knee” includes 3.458 patients with any kind of knee injuries, and the group “Non Knee” includes the remaining 26.321 patients without a knee injury. In patients with severe trauma approximately every ninth case suffered from knee injuries (n = 3458, 11.6%). Patients with knee injuries were slightly younger and had a significantly increased ISS (Table 1).

**Table 1:** Demographic data

	**Knee**	**Non Knee**	**Total**	**p-value**
**No. of patients**	3.458	26.321	29.779	-
**Gender (% males)**	71.1%	73.1%	72.8%	0.017
**Age (years)**	42.0±18.9	43.0±20.8	42.9±20.6	<0.001
**ISS**	31.0±11.9	29.7±12.8	29.9±12.7	<0.001

The p value refers to the comparison of the Knee with the Non Knee group.

**Figure 1:**
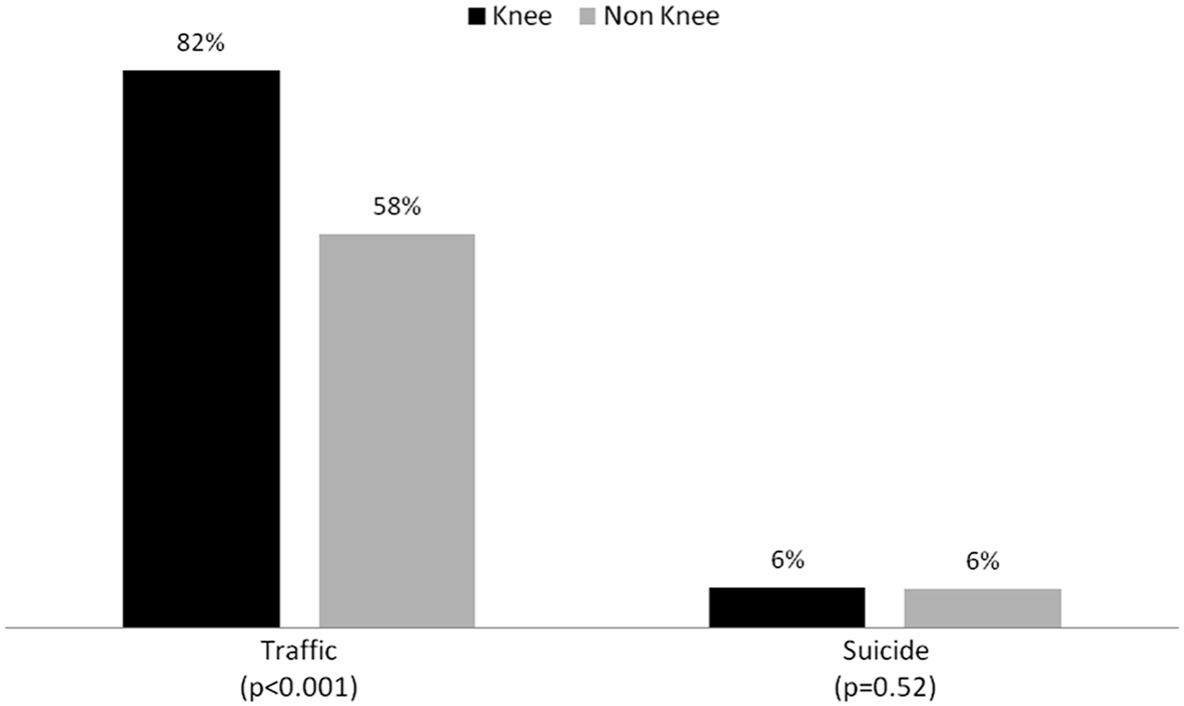
Knee injury and trauma mechanisms.

**Figure 2:**
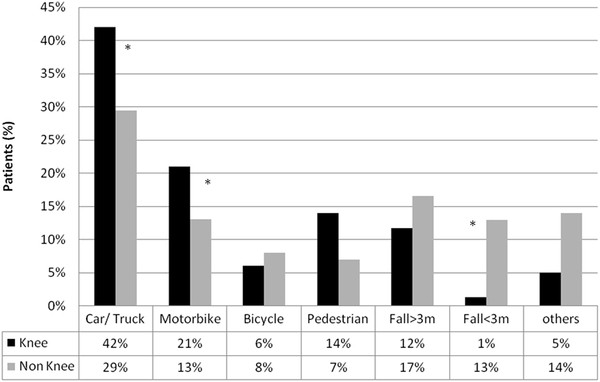
Details of injury mechanisms.

Concerning the *mechanism of injury*, knee injuries are significantly more often caused by traffic accidents compared to other reasons, e.g. suicide (Figure 1). Focusing on traffic accidents, knee injuries are more often associated with car/truck or motorbike accidents. Other traffic trauma mechanisms like bicycle or pedestrian accidents are less frequently associated with knee injuries (Figure 2). Furthermore, knee injuries seem to be seldom associated with low falls (Figure 2).

**Figure 3:**
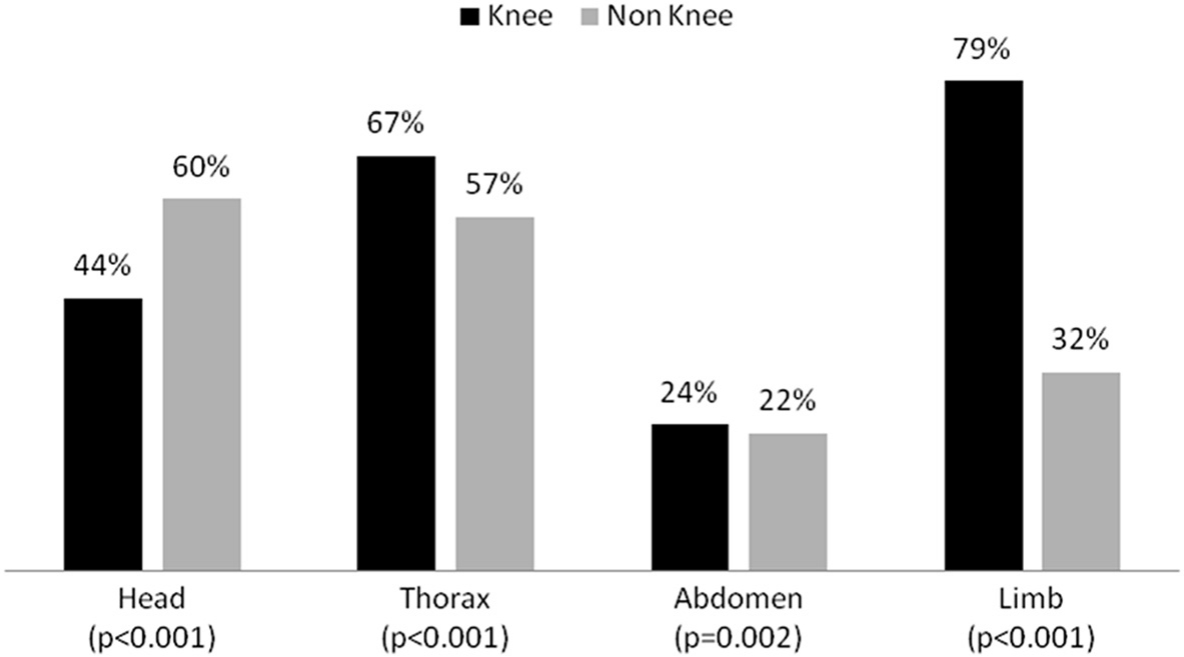
Association of knee injuries with severe (AIS severity ≥ 3) concomitant injuries of other body regions.

**Figure 4:**
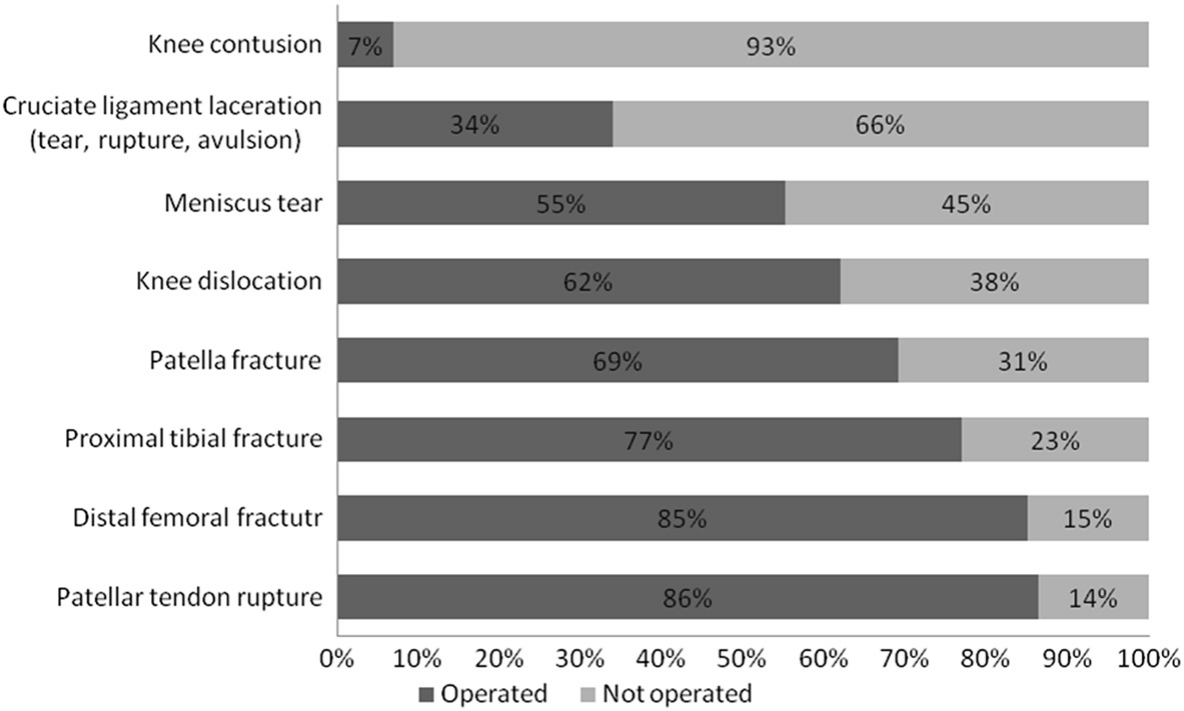
Management strategies of knee injuries.

The *injury pattern* is documented by the incidence of severe concomitant injuries (AIS ≥ 3) in different body regions (Figure 3). Knee injuries are associated with severe chest and limb injuries (p < 0.001) and are more seldom described in patients with head trauma (p < 0.001). Focusing patients with injuries of the lower extremities (n = 14,305), the knee was involved in 24.2% cases. Analyzing the distribution of the knee injuries, the three most often observed diagnoses were patella fractures, cruciate ligament tears and proximal fractures of the tibia (all prevalence rates above 30%, Table 2). Although traumatic knee dislocations are observed in only 4.4%, this injury required many surgical procedures (1.7 operations per knee dislocation, Table 2, Figure 4).

**Table 2:** Distribution of injuries of the knee region and mean number of operations per diagnosis in patients that required at least one operation

**Diagnosis**	**No. of operations per diagnosis**	**Prevalence of injury**
Patellar tendon rupture	1.5	4.1%
Patella fracture	1.3	37.1%
Knee dislocation	1.7	4.4%
Cruciate ligament tear	1.5	38.0%
Femur fracture	1.9	18.7%
Tibia fracture	2.0	32.9%
Other knee injury	1.4	16.4%

This analysis is based on 4.388 different diagnoses in the Knee group.

As far as the *outcome* of both groups is concerned, the Knee group required on average about 4 more days on ICU and 13 more days in the acute care hospital (Table 3). Accordingly, costs were significantly increased in the Knee group (40,116 vs. 25,336 Euro, respectively; p < 0.001; Table 3). Mortality was 9.3% for the Knee group and 19.3% for the Non Knee group (p < 0.001).

**Table 3:** Outcome of patients with and without knee injury

	**Knee**	**Non Knee**	**Total**	**p-value**
**Overall hospital stay (days)**	38.1 ± 33.0	25.5 ± 29.1	26.9 ± 29.9	<0.001
**ICU stay (days)**	15.2 ± 16.8	11.4 ± 14.3	11.8 ± 14.6	<0.001
**Intubation duration (days)**	9.9 ± 14.2	7.4 ± 11.6	7.7 ± 12.0	<0.001
**Mortality**	14.5%	17.4%	16.7%	<0.001
**Total costs of treatment (Euro)**	40,166 ± 28,266	25,336 ± 22,750	27,087 ± 23,951	<0.001

## Discussion

Since hospital mortality of multiple injured patients is decreasing during the past decades, the relevance of early detection and initiation of adequate treatment in patients with knee injuries increases [7]. Following the presented results, 90.7% of all patients with knee injuries were discharged alive. The majority of this population is constituted by young patients (42.0 years old), who have to deal with potential functional knee disabilities such as instability, reduced range of motion or pain for many decades. Additionally, endoprosthetic replacement in this young population presents usually only compromise and not permanent solution. Identification of patients at risk for injuries in the knee region may help to decrease late complications.

According to our data, traffic accidents with car/truck or motorbike result more commonly in knee injuries than suicide attempts or falls. A recent German accident investigation study analyzed more than 40,000 victims of road traffic accidents from 1985 to 2003 [1,2]. In accordance to our results, the authors demonstrated an increased knee injury rate in motorbike and car or truck patients. Contrary to our study, most patients were not severely injured (ISS < 16). A reduction of knee injury prevalence was observed during this period probably because of evolution in car design and the increased use of protective pads and clothes developed for motorcyclists. However, identifying the precise mechanism of knee injury in traffic accidents was not possible, as many components seemed to interact. Severe lower limb injuries of bicyclists or pedestrians are usually seen in collision with cars [13]. The most common injury is the proximal tibia fracture.

Jumps or falls from a height greater than 3 m cause usually fractures in the thoracolumbar spine, ankle and foot. Thereby, fractures of the ankle and the calcaneus are most commonly described [14]. The small incidence of knee injuries in this population may base upon three possible explanations: Firstly, as jumpers jump foot first for a suicide, there is usually no direct impact on the patella [15]. The second reason may be found in the absence of rotation-varus/ valgus forces that put into stress the collateral and cruciate ligaments. Finally, severe injuries (ISS ≥ 16) are usually induced by falls from a height less than 3 m if the person falls with the head first.

Most common injuries observed in our study are patella fractures caused by direct impact on the knee and cruciate ligament ruptures caused by rotation-varus/ valgus stress mechanisms. Although patellar tendon ruptures are seldom represented (4.1%), this injury requires surgery very often (86%). Etiology of these ruptures in young individuals without risk factors such as corticosteroid use, rheumatoid diseases or diabetes was usually direct trauma with or without open wounds in that region [16].

As far as treatment of injuries of the knee region is concerned, we observe that 38% of knee dislocations, 45% of meniscus tears and 66% of collateral and cruciate ligament injuries were treated non-operatively. Possible explanations for these unexpected results cannot be elucidated by our database. However, based on the high injury severity of the patients included we believe that potential damage control or intensive care treatment determined the decision for non-surgical treatment [17].

Referring to the main aspects of treatment duration as well as primary outcome, it has to be stated that patients with knee injuries were slightly younger, less often male gender and had suffered a higher injury severity in the present study. However, analyzing the extent of differences focusing age and gender distribution, this aspect seems actually negligibly. Nevertheless, one might argue that increased treatment durations as well as costs might result solely from the increased injury severity without impact of “knee” influences. But as the differences of injury distribution seem descriptively minor (31.0 vs. 29.7), these differences might be argued as minor clinical relevance. This argument is supported by the presented mortality demonstrating an approximately 10% higher mortality in the Non Knee group despite the decreased injury severity. In addition, a statistical difference of three points ISS has already been accepted as less clinically relevant [18]. Another aspect mentioned is found in the injury severity score itself. In patients with multiple injuries confined to a single body region, the ISS considers only one of the injuries within that region [19]. In effect, the ISS ignores all but the worst injury per body region, which could result in a considerable underestimation in case of isolated extremity injuries [19].

However, treatment durations are influenced by many factors which are certainly not elucidated entirely by the presented study, although the presented register data enables vast data analyses. Nevertheless, we feel safe to argue the knee injury as one aspect to impact treatment durations and costs beside the injury severity and demographic parameters.

In conclusion, traffic accidents are associated with a higher incidence of knee injuries than falls or attempted suicides. Severe limb and chest injuries are more commonly found in polytraumatized patients with knee injuries. At last, treatment of these patients is prolonged and costs of treatment are higher.

## Competing interests

Each author certifies that he has no commercial associations (e.g., consultancies, stock ownership, equity interest, etc.) that might pose a conflict of interest in connection with the submitted article.

## Authors’ contributions

HA and EL conceived this study designing the trial, provided statistical advice on study design, analyzed the data and drafted the manuscript. HA and EL have been contributed equally to the writing of this manuscript. RL provided statistical advice on the study design, analyzed the data and supervised the conduct of the trial and data collection. RL, CK, FH and CH conceived the study and designed the trial. FH conceived the study, designed the trial, and supervised the conduct of the trial. CH conceived the study, designed the trial, supervised the conduct of the trial and data collection, provided statistical advice on study design and analyzed the data. HA takes responsibility for the article as a whole. All authors contributed substantially to manuscript revision. All authors have read and approved the final manuscript for publication.
